# Resident cardiac macrophages: crucial modulators of cardiac (patho)physiology

**DOI:** 10.1007/s00395-020-00836-6

**Published:** 2020-12-07

**Authors:** M. Sansonetti, F. J. G. Waleczek, M. Jung, T. Thum, F. Perbellini

**Affiliations:** 1 Institute of Molecular and Translational Therapeutic Strategies (IMTTS), Hannover Medical School, Hanover, Germany; 2 Fraunhofer Institute for Toxicology and Experimental Medicine, Hannover, Germany

**Keywords:** Inflammation, Phagocytes, Macrophages, Resident cells

## Abstract

Resident cardiac macrophages (rcMacs) are integral components of the myocardium where they have key roles for tissue homeostasis and in response to inflammation, tissue injury and remodelling. In this review, we summarize the current knowledge and limitations associated with the rcMacs studies. We describe their specific role and contribution in various processes such as electrical conduction, efferocytosis, inflammation, tissue development, remodelling and regeneration in both the healthy and the disease state. We also outline research challenges and technical complications associated with rcMac research. Recent technological developments and contemporary immunological techniques are now offering new opportunities to investigate the separate contribution of rcMac in respect to recruited monocytes and other cardiac cells. Finally, we discuss new therapeutic strategies, such as drugs or non-coding RNAs, which can influence rcMac phenotype and their response to inflammation. These novel approaches will allow for a deeper understanding of this cardiac endogenous cell type and might lead to the development of more specific and effective therapeutic strategies to boost the heart’s intrinsic reparative capacity.

## Introduction

For several decades, bone marrow-derived macrophages were considered as the only large phagocytes involved in homeostasis, tissue healing, and defence against pathogens. Emerging evidence has overturned this dogma and has shown that resident macrophages (rMacs) are also fundamental players in a plethora of functions and cellular interactions both in homeostasis and in the modulation of the inflammatory response following injury and in tissue remodelling. Originating from the yolk sac or fetal liver progenitors [45], tissue rMacs inhabit various organs such as the bone marrow [58], lungs [76], liver [12], pancreas [17], brain [96], and heart [34]. Differently from circulating immune cells, rMac retain tissue-specific features. This population is made up of macrophages ontogenetically older than bone marrow-derived macrophages [95], they are evolutionarily conserved [30] and present throughout the lifetime. They can proliferate in situ and this process is exacerbated during inflammation [41]. In murine cardiac tissue, resident cardiac macrophages (rcMac) are reported to constitute up to 5–10% of the non-myocyte population, a percentage that increases dramatically following cardiac damage [50, 89]. With their peculiar spindle-like morphology, these resident immune cells take part in a large variety of physiological mechanisms which indeed include efferocytosis [26] but also immune surveillance, cardiac conduction [51, 53], bio-storage [60], cardiac regeneration [7, 62], hemodynamic interactions [72], coronary development and maturation [64]. Besides they are also immune modulators following injury or in the disease state where they orchestrate the production of both pro- and anti-inflammatory signals [28], release proangiogenic mediators [64], phagocyte apoptotic cardiomyocytes (CMs) [26] and promote or inhibit the recruitment of circulating immune cells to the injured area [10, 63]. This double function was observed in models of myocardial infarction (MI), where rcMacs could stimulate a persistent inflammatory response leading to maladaptive remodelling and, at the same time, promote tissue healing by repressing the inflammatory response [63]. To explain this paradox, scientists are currently studying the ability of rcMacs to sense various stimuli and respond by modulating their phenotype. In response to cardiac injury, rcMacs alter their gene expression profile and their surface receptors which result in a further increased heterogeneity of their phenotype. cMac plasticity is characterised by a complex polarization process that, in vitro, is often oversimplified in M1 or M2 phenotype, where M1 are considered as pro-inflammatory and M2 as anti-inflammatory macrophages [75]. However, this simple macrophage polarization paradigm does not adequately reflect the complex multicellular in vivo situation of the heart. Recent single-cell sequencing experiments revealed transcriptional differences in rMac subgroups, confirming their diversity and heterogeneity in terms of both origin and function [38]. Additionally, transcriptome analysis from Hoyer et al. discovered tissue macrophages response systemically upon remote injuries like MI, stroke or sepsis by altering tissue-specific gene expression. This result highlights the microenvironment of rcMacs could be the key to improve systemic immune reaction following injuries [52].

In this review, we summarize the state-of-the-art-knowledge of rcMacs origin, classification, and roles in the context of cardiac tissue. We also explore the potential therapeutic applications for cardiac macrophage modulation and the limitations associated with their in vivo heterogeneity and complex response. Finally, we envision how novel findings and enhanced knowledge can lead to breakthroughs in cardiovascular research which might ultimately result in innovative therapeutic strategies.

## Origin and characterization of resident cardiac macrophages

The onset of new technologies such as genetic fate mapping and lineage tracing has allowed to label and trace the cells from which rcMacs originate and to monitor their phenotypic transition during tissue development [78]. These technologies have mostly been applied to murine models and they have identified different waves of rcMac formation [78]. Distinct lineages of rcMacs exist within the ventricular myocardium of the developing heart and playing as essential regulators during cardiac development [64]. According to their cardiac localization and origin, it is possible to identify at least two distinct subsets of macrophages, CCR2^−^ and CCR2^+^ (C–C chemokine receptor type 2) [64]. CCR2^−^ cells originate from yolk sac progenitors, whereas CCR2^+^ derive from fetal monocyte progenitors, which is also reflected in their divergent gene expression profiles [64]. CCR2^−^ cells are the first macrophage population appearing in the cardiac tissue at embryonic day 12.5 (E12.5), whereas CCR2^+^ inhabits the heart at E14.5. These cells are also confined in different regions of the heart [64]. More specifically, CCR2^−^ are mostly found within the myocardial wall and in proximity to the coronary vasculature, whereas CCR2^+^ are in the trabecular projection of the endocardium [64]. These macrophages remain in the cardiac tissue for their entire life-span. For their embryonic origin and intrinsic self-renewal capacity, CCR2^−^ rMacs are also defined as “resident population”. On the contrary, CCR2^+^ subset originates from haematopoiesis and their number is ensured by recruitment of circulating monocytes. For this reason, this subset is also defined as “non-resident population” [64]. Clinically, the association of CCR2^+^ macrophages abundance on LV remodeling and cardiac function has been shown in patient with heart failure [11].

During their development and in response to different environmental stimuli and functional responses, macrophages can be activated and functionally categorized into certain subgroups including M1, or M2 phenotypes. It is important to reiterate that this classification does not appropriately depict the in vivo spectrum of macrophage sub-populations present in both the healthy and diseased myocardium. In vitro this heterogeneity is reduced and the stimulation is applied in a more controlled environment, as such this simplified definition of M1/M2 is more acceptable. M1 or “classical” activated macrophages are pro-inflammatory phagocytic cells involved in the initial stages of inflammation and this phenotype is generally attributed by infiltrating monocytes [107]. Differently, the M2 or “alternative” activated macrophages are anti-inflammatory cells implicated in the resolution of the inflammatory process [88] and normally rcMacs in steady-state heart reflect this phenotype [107]. In vitro, M1 cells are known to secrete pro-inflammatory cytokines such as nitric oxide (NO), tumor necrosis factor (TNF-α), and interleukin 12p70 (IL-12p70) thus eliciting a robust inflammatory response [110]. On the contrary, the M2 in vitro activation leads to anti-inflammatory cytokines secretion which includes transforming growth factor (TGF-β), interleukin 10 (IL-10), and arginase-1 (Arg1). These cytokines support the repression of the inflammatory response, favour tissue healing and collagen deposition [74, 110]. The M1 or M2 phenotype is not permanent and can change. It was recently reported that rcMacs (mostly M2) can transition to M1-like phenotype in aged mice [69]. The M1/M2 paradigm was not only proposed based on the activation status, but it was also confirmed by distinct metabolic profiles, alterations in cell morphology [16], gene transcription [66] and functional efferocytosis [33, 37, 47, 54].

Another important aspect of macrophage biology is the heterogeneity in origin and phenotype following cardiac injury or during tissue remodelling. In this context, several markers are efficiently used to distinguish infiltrating and rcMacs, unfortunately, they are often not consistently expressed across animal species thus complicating the translation of research findings. Transgenic animals with fluorophore-labelled macrophages [33] or Cre-loxP macrophage reporter mice [99] can be helpful to provide informative data of specific cell types and overcome technical issues associated with antibody combinations. To date, one of the most common markers to discriminate resident and non-rMacs is CCR2^−/+^ which is conserved in human, rat, and mouse [10, 34]. Other options are C-X-3-C Motif Chemokine Receptor 1 (CX3CR1) and the major histocompatibility complex class II (MHCII). Using these markers, CX3CR1^+^MHCII^−^ embryonic macrophages were identified in hearts from new-born mice and it was demonstrated how they tent to progressively diversify by increasing MHCII expression and decreasing CX3CR1 expression during aging [79]. In human, HLA-DR represents human homologue of MHC-II and human cardiac macrophages could be subdivided into three distinct subsets (CCR2^+^HLA-DR^low^; CCR2^+^HLA-DR^high^; CCR^−^HLA-DR^high^) based on CCR2 and HLA-DR [11]. Alternatively, lymphocyte antigen 6 complex locus C (Ly6C) and MHCII were also used to efficiently distinguish four distinct subgroups of murine macrophages [39, 111]. Ly6C^−^/CCR2^−^/MHCII^high^ and MHCII^low^ were shown to label macrophages deriving from the yolk sac, while Ly6C^+^CCR2^−^ and Ly6C^+^CCR2^+^ are macrophages deriving from haematopoiesis [39, 111]. In rats, Ly6C marker is replaced by CD43^high/low^ [1], whereas for human samples the equivalent marker is CD14 [11]. Recently, TIMD4 (T-cell immunoglobulin and mucin domain containing 4) and LYVE1 (Lymphatic vessel endothelial receptor 1) were identified as new markers for murine rcMacs [28].

**Fig. 1:**
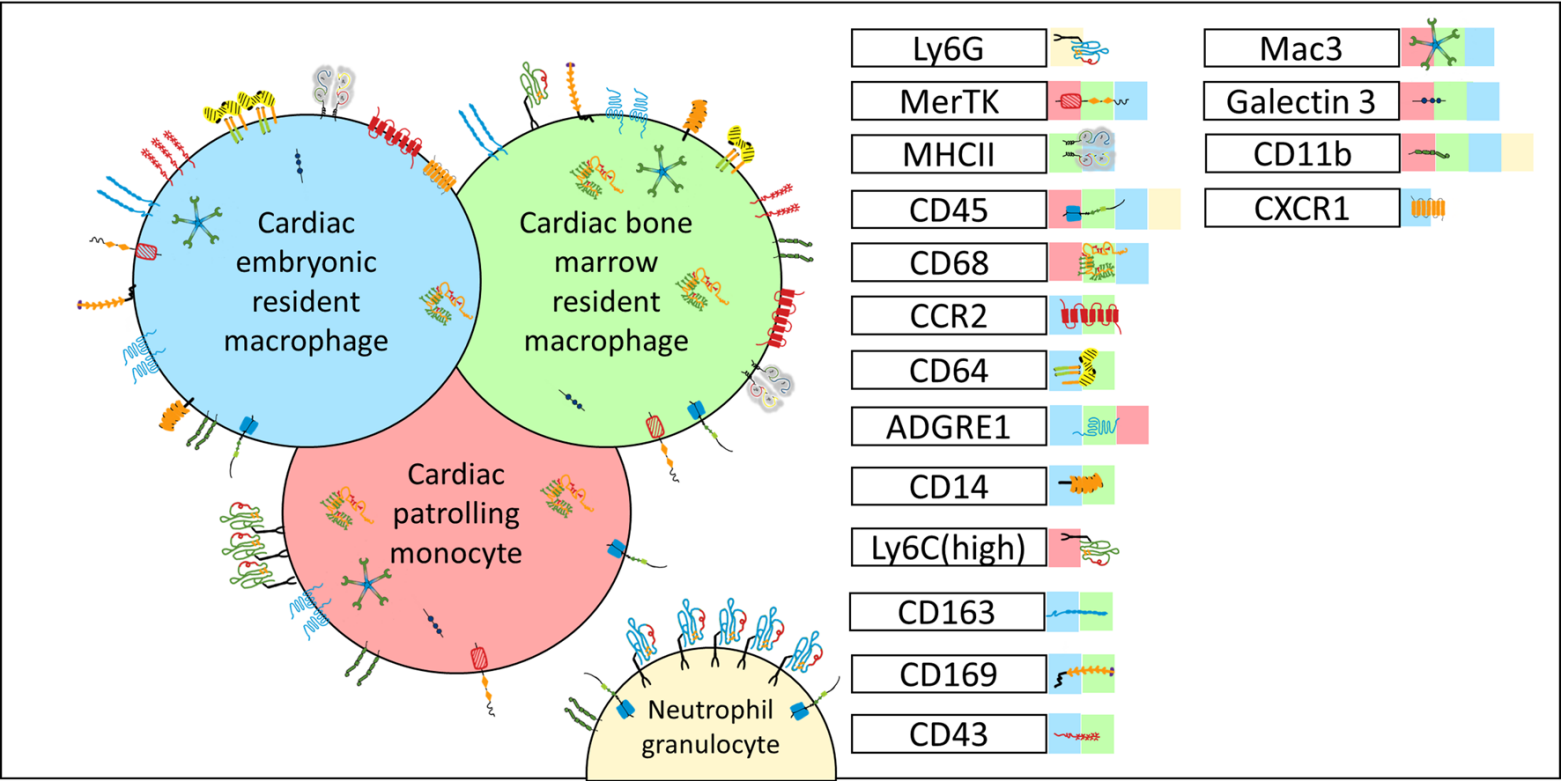
Summary of the most commonly used markers for phenotypic characterization of resident and bone-marrow-derived immune cells. The markers’ abbreviations refer to Table 1

Other common macrophage markers in human, mouse, and rat are CD68 [20, 29, 46, 112], MerTK (myeloid-epithelial-reproductive tyrosine kinase) [38], Mac-3 [70], galactose-specific lectin 3 (Galectin 3) [85] and CD163 [1, 29], these markers, however, do not discriminate between resident and non-rMacs. Other options are F4/80 [8, 105] which is mouse-specific and CD169 [29, 112] or CD64 [38] used for rat and mouse tissue [6, 98]. In human specimens, EMR1 (epidermal growth factor-like module-containing mucin-like hormone receptor-like 1) is the homolog of F4/80, and it labels both macrophages and granulocytes [4, 48]. CD11b (ITGAM) is also not sufficiently specific as it targets monocytes, neutrophils, and natural killer cells (NK cells) [70, 113]. A completely different set of markers is used to discriminate in vitro M1 and M2 macrophages. Inducible nitric oxidase (iNOS/NOS2) has been considered for several years a standard M1 marker [108], while Arg1 or CD206 were used for M2 macrophages [109]. Recent studies have identified CD38, G-protein coupled receptor 18 (Gpr18), and Formyl peptide receptor 2 (Fpr2) as more appropriate options for M1 cells, and early growth response protein 2 (Egr2) and c-Myc for M2 cells [55] (Fig. 1). In coronary artery disease (CAD) patients, it has been proven that the majority of monocyte-derived macrophages (MDMs) have a round shape compared to healthy people with a lower expression of CD206 and CD163 [32]. To facilitate the reader, the markers described in this paragraph are summarised in Table 1.

**Table 1:** Summary of markers commonly used to identify the various monocyte and macrophage subpopulations in different species

Marker name	Description	Commonly used for (human/mouse/rat)	Is used for	References
EMR1/F4/80	Epidermal growth factor-like module-containing mucin-like hormone receptor-like 1	h/m	Macrophages and granulocytes in human Pan macrophage lineage marker in mouse	[8, 105]
c-Myc	Cellular myelocytomatosis oncogene	m	Monocytes, M2 macrophages	[55]
CCR2^±^	C–C chemokine receptor type 2	h/m/r	Resident/non-resident macrophages	[10, 11]
TIMD4	T-cell immunoglobulin and mucin domain containing 4	m	Resident macrophages	[28]
LYVE1	Lymphatic vessel endothelial receptor 1	m	Resident macrophages	[28]
CD11b/ITGAM/Mac-1	Cluster of differentiation 11b/Integrin alpha M/Macrophage-1 antigen	h/m/r	Monocytes, macrophages, NK cells and neutrophils	[70, 113]
CD14^+^	Cluster of differentiation 14 GPI anchored TLR4 co-receptor	h	Classical/inflammatory monocytes, macrophages	[11, 81, 87]
CD163	Cluster of differentiation 163	h/m/r	Anti-inflammatory monocytes, macrophages, neutrophils	[1, 29]
CD169	Cluster of differentiation 16/sialoadhesin	m/r	Monocytes, macrophages	[6, 29, 98, 112]
CD206/Arg1	Cluster of differentiation 206/Arginase 1	h/m	M2 macrophages	[109]
CD38	Cluster of differentiation 38	m	M1 macrophages	[55]
CD43^high/low^	Cluster of differentiation 43/Sialophorin/Leukosialin	r	Infiltrating/resident macrophages	[1]
CD64/FcrR1	Cluster of differentiation 64 Fc gamma receptor 1A	h/m/r	Monocytes, macrophages	[6, 11, 38, 98]
CD68	Cluster of differentiation 68/Macrosialin	h/m/r	Pan macrophage lineage marker	[20, 29, 46, 112]
Egr2	Early growth response protein 2	m	M2 macrophages	[55]
Fpr2	Formyl peptide receptor 2	m	M1 macrophages	[55]
Galectin 3/Mac-2	Galactose-specific lectin 3/Macrophage-2 antigen	h/m/r	Pan macrophage lineage marker	[85]
Gpr18	G-protein coupled receptor 18	m	M1 macrophages	[55]
iNOS/NOS2	Inducible nitric oxidase	h/m	M1 macrophages	[108]
Ly6C^±^	Protein domain of Ly6 complex locus C	m	Classical (pro-inflammatory)/non-classical (patrolling) monocytes	[39, 81, 111]
Mac-3	Macrophage-3 antigen	h/m/r	Pan macrophage lineage marker	[70]
MerTK	Myeloid-epithelial-reproductive tyrosine kinase	h/m/r	M2 macrophages, phagocytes	[38, 101]
CX3CR1	C-X3-C motif chemokine receptor	h/m	Non-classical monocyte derived macrophages	[79]
MHCII	Major histocompatibility complex class II	h/m/r	Define subgroups of resident macrophages dendritic cells, B cells	[79]
HLA-DR	Human homologue of MHC-II	h	Monocytic lineage	[11]

## Role of cardiac macrophages

**Fig. 2:**
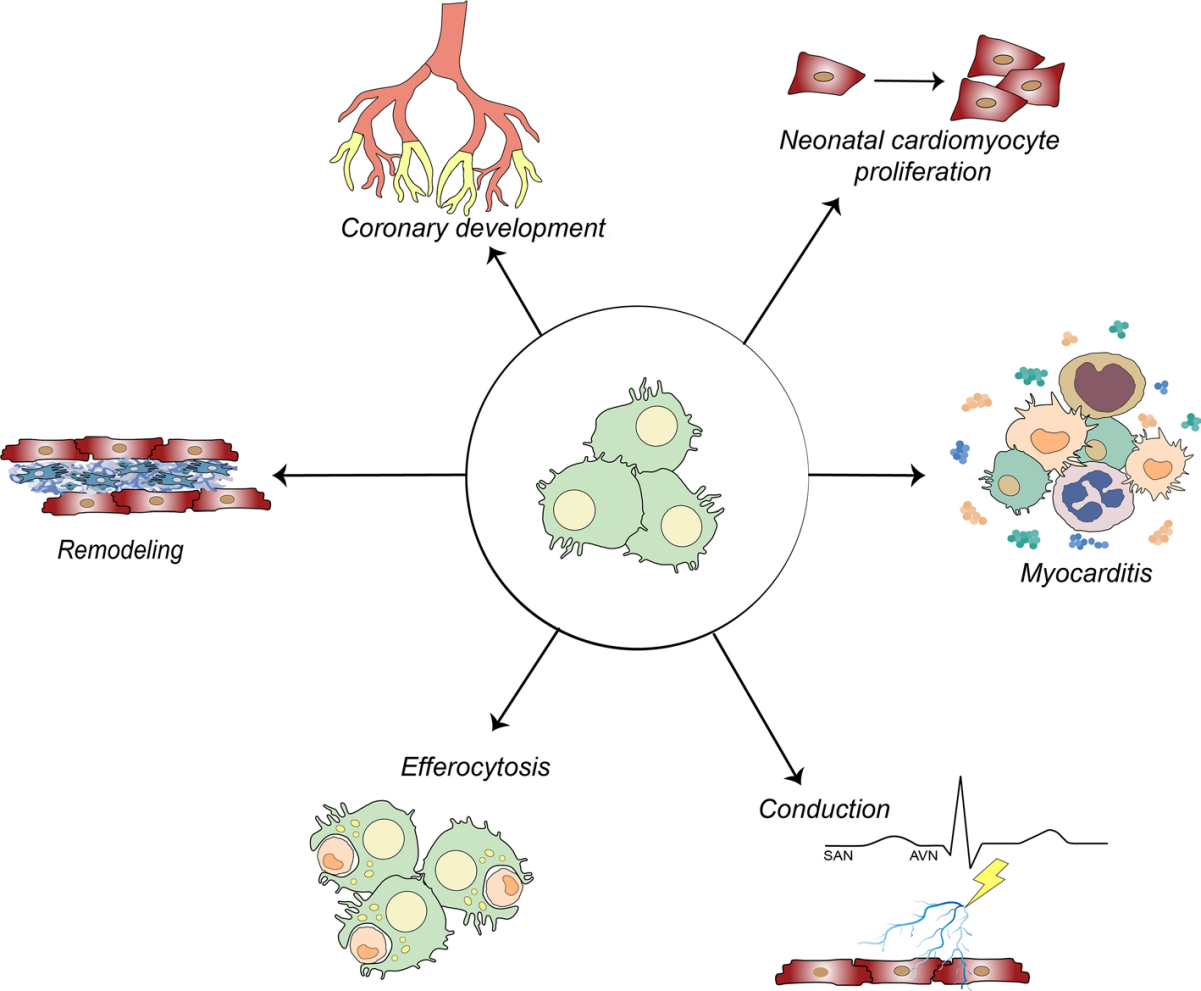
Roles of cardiac macrophages following injury. Cardiac macrophages are involved in cardiac conduction, efferocytosis MerTK-mediated, suppression of maladaptive remodelling, coronary development and maturation and neonatal cardiomyocyte proliferation (*SAN* sinoatrial node, *AVN* atrioventricular node)

Over the past decades, the understanding of macrophage functions and physiology has been revolutionized. Here, we summarize the current knowledge about rcMac functions (represented graphically in Fig. 2) in both health and disease and we highlight outstanding areas of investigation.

### Efferocytosis

Efferocytosis is the process by which apoptotic cells are removed by phagocytic cells, as such, it is one of the most important roles of rcMacs. Following MI and sudden cardiomyocyte loss, the immune system reacts and cardiac macrophages start the process of removing the necrotic cells and to contribute to efferocytosis-mediated cardiac repair. A high number of necrotic CMs and/or impaired efferocytosis activity can be responsible for an acute inflammatory response, which results in collateral cell death and might ultimately lead to maladaptive repair. Recent evidence suggested the efferocytosis process to be compromised in CAD patients. Eligini et al*.* have confirmed that, compared to healthy individuals, CAD patients have MDMs with a reduced efferocytosis capacity and pro-inflammatory features, leading to high risk of ruptures in coronary plaques [32]. Moreover, several studies have demonstrated that, in the atherosclerotic plaque, MerTK function is compromised. MerTK is a macrophage receptor that mediates the binding and phagocytosis of apoptotic cells and most of the macrophage literature is focused on MerTK related pathway. Using an ischemia–reperfusion (I/R) injury model, DeBerge et al. have demonstrated that reperfusion-induced cleavage of MerTK limits the capacity of cardiac macrophages to clear necrotic cells, impairing inflammation resolution and thus cardiac repair [26]. Mice deficient for the MerTK receptor displayed left ventricle (LV) dilatation, increased infarct size and fibrotic scar formation. The opposite was observed in mice resistant to MerTK cleavage, which presented decreased infarct sizes and enhanced cardiac function. The authors also demonstrated that bone marrow-derived monocytes have an important role in MerTK cleavage in resident cardiac macrophages [26]. In line with this study, Nicolás-Ávila et al. have confirmed that deletion of MerTK in cMacs leads to defective autophagy and compromised capacity in mitochondria removal, triggering the activation of pro-inflammatory pathways, ventricular alterations and metabolic cardiac disorders [82].

Novel findings have reported that the role of MerTk is also strictly related to CD36, a scavenger receptor involved in the phagocytosis of apoptotic CMs mediated by macrophages [27]. Using an MI model, Dehn et al*.* have shown that mice deficient for CD36, display reduced expression of the phagocytic receptor MerTK and nuclear receptor subfamily 4, group A, member 1 (Nr4a1) [27]. Mice with a double knockout (KO) for CD36 and MerTK, subjected to MI, showed increased myocardial rupture compared to wild-type mice [27]. Similarly, Nr4A is also a crucial protein necessary for phagocyte survival and for the induction of MerTK expression [27]. In line with this, in silico analysis identified the direct binding site of this protein in a MerTK genomic regulatory region [27]. Further research is needed to further expand our knowledge of the receptors involved in rcMac-mediated efferocytosis and hopefully will discover novel targets to improve therapeutic strategies.

### Conduction

In the embryos, the heart is the first organ to initiate its function and generate arrhythmic contractions while it is still developing and even before there is blood to be pumped in the circulatory system. This is possible because of the contractile activity of specialized CMs located in the sinoatrial node where the electrical impulse is generated [94]. Recently, the classic perception of CMs being the sole cellular units able to propagate the cardiac electrical impulse has been revisited. Indeed, it has been reported that the action potential propagation and CM contraction can be altered also by cell–cell interactions and communication between stromal cells and CMs [59]. Fibroblasts (FBs) play a key role in CM contraction in both physiological and pathological conditions [93]. Several in vitro studies have demonstrated the crucial role of direct contact between cardiac FBs and CMs to regulate electronic coupling [59, 86]. More recently, Hulsmans et al. were the first to demonstrate that rcMacs are also important mediators in this process and can alter electrical conduction [53]. The authors reported that, in both mouse and human, the atrioventricular node (AVN) is abundant with elongated, spindle-shaped rcMacs expressing connexin 43 (Cx43). Using parabiosis studies, they confirmed that only 1% of the circulating inflammatory cells contribute to the AVN macrophage population, corroborating the new exclusive role of rcMacs in cardiac electrical activation [53]. Using macrophage-specific Cx43 KO mice [53], the authors demonstrated that the Cx43 deletion in macrophages and the innate absence of rcMacs delays conduction through the AVN. Macrophage ablation can also alter the expression profile and drastically affect AVN function, triggering arrhythmia [53]. To investigate cell–cell communication between rcMacs and CMs, they engineered a mouse model to control the macrophage membrane potential. Mice expressing photoactivatable channelrhodopsin-2 (ChR2) were light stimulated to produce cyclical membrane depolarization which was shown to modulate CM electrophysiological properties and improved AVN conduction [53]. Using a simplified in vitro model of macrophages and IPS-derived CMs co-culture, Hitscherich et al*.* have also revealed that M2 macrophages enhance CM Ca2^+^ fractional release [51]. The presence of macrophages, independent of their pro- or anti-inflammatory subtype, causes a consistent reduction of store‐operated calcium entry response in CMs. In a study by Monnerat et al., the authors demonstrated that rcMacs can dysregulate the electrical activity of CMs, leading to lethal ventricular arrhythmias. A justification of this effect was indicated as the paracrine release of interleukin 1β (IL-1β), which induced oxidative stress in the neighbouring cells thus triggering arrhythmogenic events [80]. These findings highlighted the impacts of macrophages on CMs and vice versa. Although the experimental evidence is still limited to a restricted number of studies, the importance of macrophages in CM electrophysiology and cardiac conduction is increasing and attracting the interest of the scientific community. Further investigation is still required to address whether the reprogramming or selective targeting of macrophages could represent a possible therapeutic option for conditions with conduction disorders or to modulate the cardiac rhythm.

### Coronary development, maturation and angiogenesis

Monocyte-derived macrophages are considered the main players in angiogenesis during embryonic development or following cardiac injury. These phagocytes communicate with other cells such as pericytes, endothelial cells and vascular smooth muscle cells by paracrine signalling to modulate angiogenic events [21]. Macrophage polarization has also an effect to the angiocrine secretion profile and it influences the pro- or anti-angiogenic signals released by these cells. Indeed, in vitro M1 macrophages secrete an array of pro-angiogenic growth factors such as vascular endothelial growth factor (VEGF)-A and fibroblast growth factor (FGF)-2, while M2 macrophages release high levels of platelet-derived growth factor (PDGF)-BB, chemoattractant for pericytes and matrix metalloproteinase 9 (MMP-9) which are crucial in cardiovascular remodelling [103]. In line with this, it has been observed that Annexin A1 (AnxA1) activates the pro-angiogenic phenotype in rMac. Indeed, following MI, AnxA1 stimulates macrophages to release VEGF-A leading to new vessels’ formation. KO mice for AnxA1 show macrophages with impaired capacity to release VEGF-A and compromised cardiac functions [35].

Recent findings have identified various subsets of rcMacs and their contribution to the development of the vascular system in the heart. In this context, Leid et al*.* have characterized, in the embryonic heart, different lines of embryonic macrophages and have demonstrated their contribution in vascular maturation [64]. CCR2^−^ rMacs are modulators of coronary development and maturation, eliciting the remodelling of the primitive coronary plexus through the selective expansion of perfused coronary vasculature. CCR2^+^ macrophages seem also to be involved in heart development, however, further studies to better investigate their contribution and role are needed [64]. The pro-angiogenic effect of CCR2^−^ macrophages has also been verified in vitro, where conditioned media obtained from this macrophage population could promote coronary endothelial cell migration and tube formation [64]. The pro-angiogenic capacity of CCR2^−^ macrophages was also demonstrated at the mRNA and protein level, with higher concentrations of insulin-like growth factor (IGF1) in conditioned media from embryonic CCR2^−^ macrophages than CCR2^+^. Additionally, the supplement of an IGF1 receptor-specific inhibitor was sufficient to eliminate the ability of CCR2^−^ macrophages conditioned media to prompt coronary endothelial cell migration and tube formation. These findings indicate IGF as a potential pro-angiogenic signal by which CCR2^−^ embryonic macrophages can modulate coronary development [64].

Given the evidence about the key role of rcMacs in coronary growth and development, it would be tempting to elucidate the potential function of CCR2^+^ in heart development as well as investigate how rcMacs can influence the vascular remodelling in different pathological conditions.

### Regulation of cardiomyocyte regeneration

Cardiac regeneration remains a great promise for cardiovascular research. In contrast to adult mammalians, salamander and zebrafish retain an excellent regenerative capacity and following tissue injury they can repair complex structures such as the brain and heart [44, 102]. Increasing evidence seems to indicate that macrophages are key players in this process. When myocardial damage is performed by cryoinjury to zebrafish and salamander’s hearts, they respond with inflammation, edema and collagen deposition which closely resemble the process occurring in the mammalian adult heart [102]. Despite this similarity, cardiac regeneration eventually succeeds by a fine-tune cooperation between pro-fibrotic and pro-regenerative pathways which are mediated by macrophages. The loss of macrophages during the initial phases of tissue injury results in the interruption of cardiac regeneration and impaired recovery [43]. Using macrophages with a genetic deletion for collagen type IV alpha-3-binding protein (col4a3bpa), Simões et al. demonstrated that macrophages are directly involved in collagen deposition during zebrafish heart regeneration [102]. Recent evidence revealed that cardiac macrophages are also key drivers in the regenerative and reparative response of injured adult rodent hearts. Neonates can recover after apical resection [90] or MI [91], with minimal hypertrophy or fibrosis, this regenerative capacity, however, is lost within 7 days after birth. On the opposite, following cardiac injury, the adult heart undergoes cell death, acute inflammation and scar formation which eventually lead to tissue remodelling and heart failure (HF). Aurora et al. noticed that the immune response of postnatal day 1 (P1) and P14 mice is different and they identified significant alterations in several immune cells involved in the reparatory process, particularly in macrophages. Using a macrophage depletion model, the authors demonstrated that these phagocytes are indispensable for neonatal heart regeneration and angiogenesis in P1 mice. Indeed, following MI, neonatal mice with macrophages’ depletion lose their capacity to regenerate myocardium, with increased collagen deposition and scar formation [7].

Lavine et al*.* also reported the direct implication of rcMacs in the regenerative process [62]. Following cardiac injury, the neonatal heart rcMacs respond by inducing minimal inflammation and activating tissue repair by boosting coronary angiogenesis and CM proliferation [62]. On the contrary, in the adult injured heart, rcMacs are replaced by pro-inflammatory macrophages and monocyte-derived macrophages which induce inflammation and oxidative stress, with an inadequate capacity to promote cardiac repair. In line with this, inhibition of monocyte recruitment after adult cardiac injury preserves rMacs population, suppresses inflammation, and leads to adult cardiac repair [62]. A higher number of CCR2^−^ cardiac macrophages were observed in the hypoxic condition, which promotes CM proliferation in newborn human and animal models. This reinforces the hypothesis of the potential role of rcMacs in the regulation of CM proliferation [67]. Over the last decades, transcriptomic and epigenomic analyses have confirmed that the inflammatory response mediated by embryonic macrophages play a crucial role in cardiac regeneration. On this topic, Wang et al*.* reported differences in the immune response in regenerative and non-regenerative hearts following MI [106]. The regenerative process is triggered by a unique immune response which involves chemokine C–C motif ligands 4 (CCL4), a macrophage-secreted factor, and insulin-like growth factor 2 mRNA-binding protein (IGF2BP3), encoding for an RNA-binding protein [106]. CCL4 is preferentially expressed in P1 macrophages rather than in P14, highlighting how the neonatal heart regeneration is governed by the embryonic cardiogenic gene program [106].

Despite the precise role played by resident and non-resident macrophages in the reparative process of the injured heart, there is a growing recognition that these immune cells might represent the turning point for the identification of new mechanisms modulating CM regeneration in response to injury.

### Adverse LV remodeling and dysfunction

Considering the important role associated with intercellular connections, paracrine factors’ release and collagen secretion, it is not surprising that macrophages are essential regulators of LV maladaptive remodelling as demonstrated in various research models. The transcriptomic analysis, performed by Simões et al., identified a different expression pattern in collagens and ECM genes sets in macrophages isolated from the damaged heart of zebrafish and mouse, suggesting their direct role in scar formation [102]. Similar findings were confirmed by Ma et al*.* where macrophages were indicated as the main driver of cardiac fibrosis both in vivo and in vitro [68] by boosting the production of interleukin (IL)-6 from cardiac FBs. This was connected to TGF-β1 release and small mother against decapentaplegic (Smad3) phosphorylation enhancing the activation of cardiac FBs into myofibroblasts [68]. Similarly, Shahid et al*.* have confirmed that, during HF, the accumulation of monocyte-derived macrophages in the damaged cardiac tissue is associate to collagen deposition and transition of FBs into myofibroblasts, ultimately resulting in severe cardiac remodeling [97]. A recent study by Dick et al., reported that within the infarct zone, cardiac rMacs account for approximately 2–5% of the total macrophages during the early stages of cardiac damage. Their depletion, however, leads to defective cardiac function, inducing pathological remodelling and LV dysfunction [28]. The justification for such specific behaviour has been proposed by a small cluster of genes exclusively expressed by human and murine rcMacs which include *TIMD4*, *LYVE1,* and *IGF1* [28]. Recent studies have shown that rMacs are also important in cardiac recovery after tissue injury. Depletion of neonatal macrophages, by liposomal clodronate, elicits cardiac chamber dilatation, CM hypertrophy, and interstitial fibrosis, emphasizing once again the relevance of these resident immune cells [62]. Moreover, in a murine cryoinjury model, the non-selective depletion of macrophages, by clodronate-containing liposomes, during the first week after injury resulted in impaired wound healing and higher mortality rate due to increased LV dilatation and impaired scar formation [3]. Taken together these findings clearly indicate the beneficial role of these resident cardiac phagocytes during tissue remodelling. More research on the topic is likely to soon identify specific mechanisms and cellular interaction that can be exploited to regulate the remodelling process ultimately improving tissue function.

### Myocarditis and rcMacs

Particularly interesting and controversial is the role of rcMacs during myocarditis or inflammatory cardiomyopathy. This pathological condition has mostly viral origins but it can also be caused by drugs, heavy metals or by deregulation of the immune system (i.e. autoimmune myocarditis) [63]. Viral myocarditis is the most frequent, with viral genomes detected in 35–70% of patients with dilated or chronic cardiomyopathy [31, 73]. This condition is characterized by the release of double-stranded viral RNA which drives the release of pro-inflammatory cytokines and the activation of different immune cells including monocytes and macrophages. The release of these chemical signals results in the alterations of cardiac tissue homeostasis, and it often evolves in adverse cardiac remodelling and occasionally in heart failure. The precise role and contribution of rcMacs in myocarditis remains unclear, however, all myocarditis in vivo models report a clear expansion of macrophage numbers. Ex vivo experiments have demonstrated that CCR2^−^/MHCII^high^ macrophages act as antigen-presenting cell for T-helper cells activation [34]. On the contrary, CCR2^−^/MHCII^low^ macrophages have a reparative phenotype, they promote CM proliferation and angiogenesis [7, 62]. In a study performed with a mouse model of myocarditis induced by encephalomyocarditis virus, it was shown that the specific depletion (clodronate-mediated) of rcMacs caused an increase in animal mortality in the acute phase [42, 77]. Different outcomes were noticed if the deletion of rcMacs was occurring in the advanced stages of the viral infection. Using a mouse model of experimental immune myocarditis, the injection of clodronate-loaded liposomes was performed during the chronic phase of the cardiac infection. The treatment lead to a reduction of rcMacs which had beneficial effects in cardiac function and reduced maladaptive tissue remodelling [63]. Similarly, in a murine model of Coxsackievirus B3 myocarditis, it was shown that in response to the viral infection, macrophages secrete galectin 3 involved in the formation of fibrotic tissue following viral myocarditis [42]. Indeed, the depletion of macrophages as well as the pharmacological inhibition of galectin 3 resulted in a reduction of the acute inflammation and deposition of fibrotic tissue [42]. Emerging evidence also indicate that the de-regulated or the maladaptive inflammatory response can have a negative impact on the progression of myocarditis induced tissue damage. Several receptors were shown to be involved in the transition from acute to chronic inflammation. On this topic, toll-like receptors (TLRs), a family of receptors active in the immune response against pathogens are studied the most. During viral myocarditis TLR3 was shown to bind the viral RNA thus inhibiting the replication of the virus. Mice affected by inflammatory cardiomyopathy CVB3 mediated with a deletion in TLR3 are more susceptible to develop lethal myocarditis.

Doxorubicin, a drug used to treat cancer and known for its cardiotoxic effects, can also cause myocarditis. Mice with a deletion of TLR4 and TLR2 display a reduced cardiotoxicity following doxorubicin treatment, confirming the important role that these receptors play in this process [83, 92].

A better understanding of the role of this cardiac population might have a potential impact for the treatment of infectious diseases which are known to induce myocarditis or that induce a systemic inflammatory response. This is the case of various viral infections including the most recent SARS-CoV-2 which is currently having enormous socio-economic effects on the worldwide public health and economy. Lung monocytes and macrophage response during SARS-CoV-2 infections has recently been described and the contribution of rcMacs is likely to soon be investigated to develop potential therapeutic interventions to attenuate macrophage-related inflammatory reactions [56].

## Macrophage-mediated therapeutic strategies

**Fig. 3:**
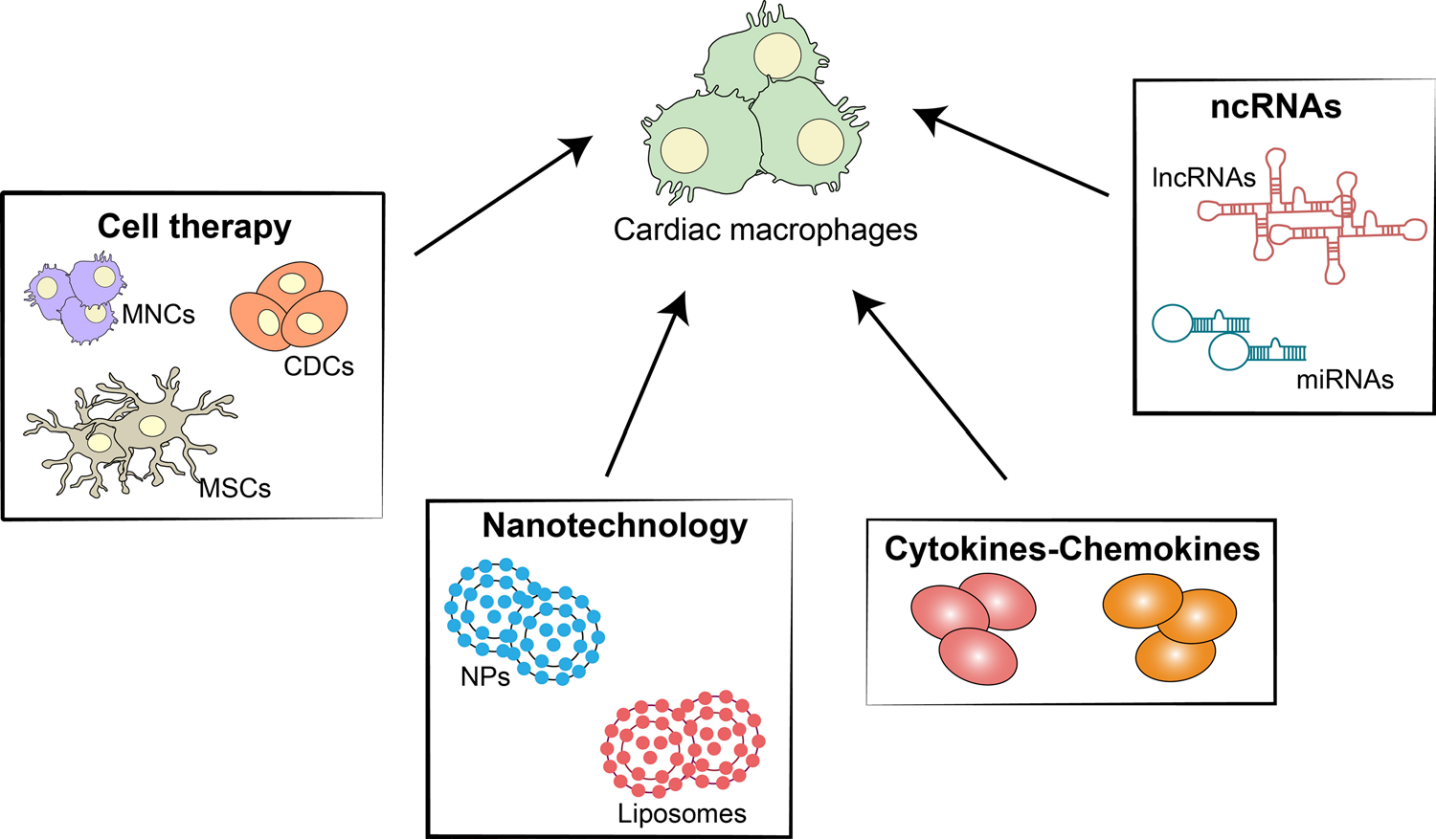
Therapeutic approaches to target cardiac macrophages. The most used macrophage-mediated therapeutic strategies are cell therapy, application of nanotechnologies, cytokines/chemokines modulation and non-coding RNAs (*MNCs* bone marrow mononuclear cells, *CDCs* cardiosphere-derived cells, *MSCs* mesenchymal stromal cells, *NPs* nanoparticles, *ncRNA* non-coding RNAs, *lncRNAs* long non-coding RNAs, *miRNAs* microRNAs)

The majority of therapeutic strategies for CVDs are targeting bone marrow-derived macrophages. Whereas rcMacs heterogeneity and complexity are still less appreciated, which makes them as a novel direct or indirect target for therapeutic applications (Fig. 3).

While pivotal role of cardiac macrophages in cardiac development as well as in diseases have led to rise of attention, strategies for specific cardiac macrophages targeting still remained challenging. For this reason, despite of the extremely promising results from experimental models, most of clinical attempts did not meet the expectations of the pre-clinical data. Therefore, a comprehensive overview of rcMacs dynamics and corresponding therapeutic approach is urgently needed to limit off-target effects. One encouraging strategy is the use of nanoparticles (NPs) for the treatment of CVDs. In a recent mouse study by Aouadi et al*.* NPs were enriched with siRNAs able to target macrophages and to inhibit TNF-α and IL-1β production by Map4k4 silencing [5]. Flores et al*.* have also proposed an approach to targeted macrophages with NPs in atherosclerotic plagues. The treatment induced a reduction of pro-inflammatory cytokine release and prevent the atherosclerosis progression by disrupting the CD47-SIRPα (signal regulatory protein-α) signalling [36]. A different experimental approach applied by Getts et al. where immune-modifying microparticles (IMP) were used following cardiac reperfusion injury [40]. After IMP treatment, the authors observed a decreased number of macrophages as well as a reduction in cardiac inflammation and improved cardiac function [40]. Scientific evidence seems to indicate that the pharmacological modulation of rcMacs in acute ischemia might be particularly challenging. It has been recently described that, in mice subjected to stroke, the survival time of these cells is just 20 h and they are then replaced by splenic monocytes [65]. However, although most of the resident macrophages are replaced by monocytes, a less number of these cells remain in the heart and keep self-replication, playing a crucial function [28].

The mechanisms that regulate the shift of cardiac macrophages from a pro-inflammatory (M1) to an anti-inflammatory (M2) phenotype have also been identified as novel opportunities for therapeutic intervention. Mesenchymal stromal cells (MSCs) are active players in the M1–M2 phenotypic transition [15]. As such, MSC treatment following MI was shown to promote this phenotypic switch both in vitro and in vivo where it also improved LV remodelling and function [14]. Cardiac stem cell therapy could be a practical option to selectively activate macrophages. Vagnozzi et al*.* have observed that, following an ischemia–reperfusion injury, the intracardiac injection of bone marrow mononuclear cells (MNCs) promotes the recruitment of CX3CR1^+^ and CCR2^+^ macrophages in the damaged cardiac tissue [104]. This MNC population alters function of cFBs driving to a decreased fibrotic tissue deposition, which lead to improvement in the cardiac function [104].

Intriguingly, the polarization of macrophages can also be controlled at the genic level with the aid of NPs which might provide an alternative and more precise treatment option. By silencing the interferon regulatory factor 5 (IRF5) with the help of NP-delivered short interfering RNA (siRNA), Courties et al. succeeded in the reprogramming the macrophage phenotype. The delivery of these siRNA-enriched NPs induced a reduction of M1 markers and the resolution of inflammation with improved infarct healing [23]. Bagalkot et al*.* have reported the in vitro capacity of hybrid lipid-latex (LiLa) NPs to be selectively taken up by M1 macrophages [9]. By loading LiLa NPs with an anti-inflammatory drug, they observed a reduction in the expression of pro-inflammatory cytokines in targeted M1 macrophages [9]. NPs have been also used to prevent the progression of inflammatory diseases by reprogramming the macrophage phenotype. In a study by Jain et al*.,* the authors developed NPs which able to transport IL-10 in the inflamed environment eliciting the shift from pro-inflammatory to anti-inflammatory macrophages [57]. In a MI model, Bejerano et al*.* reported the capacity of NPs, which were loaded with miR-21, to target M1 macrophages reprogramming their phenotype in anti-inflammatory macrophages, thus leading to angiogenesis, reduction in CM apoptosis and improvement in the cardiac function [13]. Similarly, Alvarado-Vazquez et al*.* induced a macrophage phenotype switch from M1 to M2 by applying NPs which overexpress CD163 in human primary macrophages [2]. Taking advantage of phosphatidylserine (PS), a ligand presents on the surface of apoptotic cells, Harel-Adar et al. engineered liposomes conjugated with PS which are recognized and phagocytised by cardiac macrophages driving the switch in the secretion of anti-inflammatory cytokines and repressing the release of pro-inflammatory mediators [49]. In this study, rats were subjected to acute MI and injected with PS liposomes. The results demonstrated decreased expression of pro-inflammatory markers such as TNF-a, and CD86 and higher expression of anti-inflammatory markers such as CD206, IL-10, and TGF-b, which indicated that the shift from pro-inflammatory to reparative macrophages has occurred. In line with this, from a whole tissue perspective, rats exhibit enhanced cardiac function, reduced fibrosis, and increased angiogenesis [49].

Cardiosphere-derived cells (CDCs) have also been used to modulate macrophage phenotype. Using an I/R rat model the authors demonstrated a cardioprotective effect of these cells which resulted in decreased apoptosis and scar formation [25]. Furthermore, the beneficial effects of CDCs were completely abolished the depletion of the systemic macrophages by the clodronate’s administration [25].

The main cause of cardiac macrophages’ polarization is strictly associated with the release of inflammatory mediators. The capacity of interleukin (IL)-4 to drive the polarization of macrophages towards the reparative phenotype has been tested in vivo by intraperitoneal injection in mice subjected to MI [100]. The treatment resulted in a higher percentage of M2 macrophages in the tissue which had beneficial effects on fibrotic tissue remodelling which prevents ruptures in the injured cardiac wall and consequently increased survival and improved cardiac function [100]. Opposite effects, however, were reported in Tribbles Psuedokinase (TRIB1)-deficient mice where the selective depletion of M2 macrophages resulted in decreased scar formation, recurrent cardiac rupture, and a higher mortality rate [100].

Non-coding RNAs (ncRNAs) are also promising tools for therapeutic strategies. There are various types of ncRNAs and they are normally classified accordingly to their size or mechanism of action. They all have important regulatory functions and they are involved in various cellular processes in both health and disease state. In the context of cardiac rMacs, recent data originated from human heart tissue have reported important differences in the ncRNAs expression profile of bone marrow-derived and embryonic macrophages [11]. Various subpopulations of cardiac rMacs carry MHCII [11] which enhances their ability to present antigens and act as antigen-presenting cells (APCs) which is crucial during both inflammation and resolution. Autophagy, a lysosomal catabolic process to dispose of organelles and cytoplasmic content, is also essential during the initial phases of immunity APCs mediated [61]. The lncRNA expression profile of macrophages undergoing autophagy was recently studied and a pathway involving metastasis-associated lung adenocarcinoma transcript 1 (Malat1) identified it as a promoter of autophagy [71]. A deeper understanding of Malat1-mir-23-3p-Lamp1 (lysosomal-associated membrane protein 1) interactions could help to further understand the role of macrophages and their role in inflammation. Another important feature of ncRNAs is the possibility of being transferred via exosomes. In this process, nanosized lipid bilayer vesicles, enriched with ncRNAs are secreted and taken up by recipient cells. Keeping in mind the regulatory function of ncRNAs, exosomal transfer provides another opportunity for cellular communication. CDCs can release exosomes which are particularly enriched in Y RNA fragment (EV-YF1) [18]. These EV-YF1-enriched exosomes target macrophages leading to an increase in the production and secretion of IL-10 [18]. In a co-culture of CMs and macrophages*,* it was observed that the overexpression of EV-YF1 in macrophages, results in a cardioprotective outcome through IL-10 secretion [18]. In vivo, rats subjected to MI and treated with EV-YF1 displayed a reduction of the infarct area, highlighting the cardioprotective effect of this Y RNA fragment [18]. Similarly, exosomes secreted by CDCs can be enriched with different miRNA including miRNA-181b which were shown to induce macrophage polarization towards a cardioprotective phenotype with associated beneficial effect at a tissue level [24]. miR-155 is overexpressed in cardiac macrophages. In a mouse model of myocarditis, this specific microRNA was highly expressed by infiltrating macrophages [22]. The systemic knockdown of miR-155 leads to reduced infiltration of monocyte-derived macrophages and reduced cardiac damage [22]. In line with this, the pro-inflammatory role miR-155 has been confirmed also in a pressure-overload mouse model [84]. Mice with a deletion of miR-155 in macrophages show reduced hypertrophy and inflammation [84], suggesting its potential for therapeutic applications. Differently, lncRNA-Macrophages M2 polarization (MMP2P) is upregulated in M2, but not in M1 macrophages [19]. Moreover, the knockdown of lncRNA-MMP2P inhibits the polarization of macrophages towards the M2 phenotype by decreasing the phosphorylation of signal transducer and activator of transcription 6 (STAT6) [19].

## Conclusions

Recent technological developments and contemporary immunological techniques are offering new opportunities to identify and study the roles and contribution of rcMac in respect to recruited monocytes and other cardiac cells. These novel approaches have already allowed scientist to better understand rcMac origin, phenotypic profile and their functional contribution in myocardial function. Basic and pre-clinical studies which involve the use of drugs or non-coding RNAs also demonstrated the potential of rcMac to regulate cellular interactions thus suggesting their use to modulate and potentially prevent tissue remodelling. The emerging evidence is also highlighting the detrimental effects induced by uncontrolled responses of this cell type. The future of macrophage-modulated therapy will have to take advantage of the mechanistic pathways that coordinate tissue repair and exploit them to develop more precise and effective therapeutic strategies.
